# Supplementary material for: Deletion of wfs1 Impairs Oligodendrocyte Precursor Cells Dorsal Distribution and Myelination Through the wfs1-hmgcs1 Axis in Zebrafish
Source: Biology (Basel). 2026 Aug 16;15(16):1402. doi: 10.3390/biology15161402 (PMC13510151; doi:10.3390/biology15161402)
Supplement: Supplementary file 1 [file biology-15-01402-s001.zip › File S1-Transcriptome sequencing data and differentially expressed genes (DEGs) in wfs1-- zebrafish at 5 dpf/2.KEGG/wfs1KOvsWT/UP/src/dre00350.html]

dre00350


- dre:116517

- Up regulated genes

116517(5.15873267159645)

- dre:30505

- Up regulated genes

30505(4.64526515149742)

- dre:541429

- Up regulated genes

541429(1.54037114709183)

- dre:445287

- Up regulated genes

445287(1.98523130799607)
- dre:394142

- Up regulated genes

394142(1.09942837424348)

- dre:561410

- Up regulated genes

561410\_1(1.20432290851387)

- dre:541429

- Up regulated genes

541429(1.54037114709183)

Close
